# Clinical outcomes and survival following placement of self‐expandable metallic stents for central airway stenosis and fistula

**DOI:** 10.1111/1759-7714.13707

**Published:** 2020-11-12

**Authors:** Katsuo Usuda, Shun Iwai, Aika Yamagata, Yoshihito Iijima, Nozomu Motono, Yutaka Takahara, Shohei Shinomiya, Taku Oikawa, Shiro Mizuno, Hidetaka Uramoto

**Affiliations:** ^1^ Department of Thoracic Surgery Kanazawa Medical University Ishikawa Japan; ^2^ Department of Respiratory Medicine Kanazawa Medical University Ishikawa Japan

**Keywords:** central airway stenosis (CAS), central airway fistula (CAF), lung cancer, esophageal cancer, self‐expandable metallic stents (SEMS)

## Abstract

**Background:**

Self‐expandable metallic stent (SEMS) placement is an urgent procedure for patients with malignant central airway stenoses (CASs) and central airway fistulas (CAFs). The aim of this study was to determine the outcome and survival after SEMS placement in patients with malignant CASs and CAFs.

**Methods:**

SEMSs were inserted into 20 patients with malignant CASs and four with malignant CAFs. Hospital records, the modified Medical Research Council dyspnea scale (mMRC) grade, performance status (PS), symptoms, procedure‐related complications and survival after placement were retrospectively reviewed.

**Results:**

Spiral Z stents were inserted in nine patients, covered Ultraflex stents in 14, and a bare Ultraflex in one patient. After SEMS placement, 20 patients (83.3%) showed improvement in mMRC grade, 19 (79.2%) showed improvement in PS, and 21 (87.5%) showed improvement in symptoms. There were three patients whose stents migrated out of place, but there were no patients with obstructive granulation, infection, or mucous plugs. Median survival days after stent insertion was 98 days for CAS and 103 days for CAF, and mean survival days was 383 ± 707 days for CAS and 93 ± 33 days for CAF. Two patients with CAS by malignant lymphoma and thymic cancer survived more than six years because they were also treated with efficient therapies. The five‐year survival rate after stent insertion was 7.7%.

**Conclusions:**

SEMS placement for CAS and CAF is associated with improvement in mMRC grade, PS and symptoms in 87.5% of patients. Patients with a malignant CAS are usually terminal, but the possibility of increasing survival rate will become a reality with new efficient therapies.

**Key points:**

**Significant findings of the study:**

- Reasonable clinical outcomes and improved survival of patients following SEMS placement for thoracic malignancy with central airway stenosis and fistula.

**What this study adds:**

- The possibility of increasing survival rate will become a reality with new efficient therapies.

## Graphical abstract

SEMS placement improved the quality of life (QOL) in patients with malignant central airway stenosis and tracheobronchial fistulas. Patients with a malignant airway stenosis are usually terminal, but the possibility of longer survival rates will become a reality with new efficient therapies.

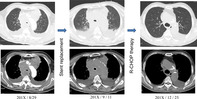

## Introduction

Malignant central airway stenoses (CASs) and central airway fistulas (CAFs) are usually managed by the placement of self‐expandable metallic stents (SEMSs), but with conflicting results. SEMSs were originally developed by Wright *et al*.^1^ in 1985. This device was first used in 1986 by Wallace *et al*.^2^

SEMS placement is a reasonable treatment option for patients with malignant CAS and CAF. However, the long‐term efficacy and safety of this treatment in patients with malignant CAS and CAF is unclear. Although patients with malignant CAS and CAF are treated as terminal, efficient therapies would be able to extend their survival.

The purpose of this study was to identify the clinical outcomes and prognosis of patients with SEMS for malignant CASs and CAFs. Furthermore, based on our data, we outline additional efficient therapies that will be crucial for improving prognosis of these patients in the future.

## Methods

### Enrolled patients

During the period between 2001 to 2019, after explaining the risks and benefits of the placement of airway stents to patients suffering from central airway stenoses or tracheobronchial fistulas (tracheoesophageal fistula, bronchoesophageal fistula, tracheomediastinal fistula), informed consent was obtained from each participating patient and/or their family prior to the procedure. SEMSs were inserted into malignant airway stenosis in 20 patients and malignant tracheobronchial fistulas in four patients (Table 1). The institutional ethical committee of Kanazawa Medical University approved this retrospective study (approved No. I487). There were 12 patients with lung cancer, nine with esophageal cancer, one with malignant lymphoma, one with thymic cancer, and one with metastasis of renal cell carcinoma. There were nine patients with stage III malignant tumors, and 15 with stage IV malignant tumors. There were 18 males and six females, with a mean age of 65.9 years (range, 47 to 81 years). We followed‐up the patients in the study for two years after stent placements. If patients survived for more than two years, they were followed‐up until 10 years after surgery. Out of 24 patients, there were three patients (two stenosis, one fistula) with acute respiratory failure. We retrospectively reviewed hospital records and procedure notes in order to extract the following data: the modified Medical Research Council dyspnea scale (mMRC) grade,^3^ performance status (PS),^4^ symptoms, procedure‐related complications and survival days after stent placement.

**Table 1:** Patient characteristics

		CAS	CAF	Total
Diagnosis	Lung cancer	11	1	12
	Esophageal cancer	6	3	9
	Malignant lymphoma	1	0	1
	Thymic cancer	1	0	1
	Metastasis of renal carcinoma	1	0	1
Stage	Stage III	7	2	9
	Stage IV	13	2	15
Types of SEMS	Spiral Z stent	9	0	9
	Covered Ultraflex	10	4	14
	Bare Ultraflex	1	0	1
Location	Trachea	10	2	12
	Tracheal bifurcation	3	1	4
	Left main bronchus	4	1	5
	Right main bronchus	3	0	3
Survival days after stent replacement	Median	98	103	101
	Mean ± S.D.	383 ± 707	93 ± 33	312 ± 630

CAF, central airway fistula; CAS, central airway stenosis.

### Bronchoscopy procedure

Flexible bronchoscopic interventions with fluoroscopic guidance were performed in endoscopy care units or operating theaters under local anesthesia with 1% xylocaine solution and conscious sedation with intravenous midazolam (2.5–10 mg), or under general anesthesia. Spiral Z stents (Cook Inc., Bloomington, IN) and Ultraflex (Boston Scientific, Natick, MA, USA) were implanted based on the condition of the airway. Each patient underwent a flexible bronchoscopy (Olympus BF‐200, BF‐240, BF‐260; Olympus, Tokyo, Japan) through an endotracheal tube. The bronchoscope was navigated to the proximal end of the lesion. Under fluoroscopic control metal markings were placed on the skin surface at the proximal and distal end of the lesion. A guidewire was inserted through the bronchoscopic channel and passed through the lesion, and the bronchoscope was then removed. A delivery catheter (Boston Scientific, Natick, MA, USA) was advanced over the guidewire to release the stent into the lesion between the proximal and distal end of the lesion. Spiral Z stents were inserted via the same method. After that, the bronchoscope was reintroduced into the airway and the position of the stent was checked. If stent repositioning was required, biopsy forceps (FB‐15C‐1; Olympus, Tokyo, Japan) were used to hold the end of the stent and the position of the stent could be adjusted. Balloon dilations inside the stent could be added. All patients were monitored with pulse oximeters and electrocardiograms (ECG).

### Statistical analysis

The computer software program StatView for Windows (Version 5.0; SAS Institute Inc. Cary,0020007AxNC, USA) was used for statistical analysis. The data is expressed as the mean ± standard deviation. A two‐tailed Student *t*‐test was performed for the comparison of data. The Kaplan‐Meier method was used to calculate the survival rate using death from any cause with a 95% confidence interval (CI), and the log‐rank test was used to compare the survival curves. A *P*‐value of <0.05 was considered statistically significant.

## Results

Before 2007 spiral Z stents were inserted in nine patients. After 2007, covered Ultraflex stents were inserted in 14, and a bare Ultraflex stent in one patient. Their locations were at the trachea in 12, at the tracheal bifurcation in four, at the left main bronchus in five, and at the right main bronchus in three patients. Four patients had airway fistulas, two had tracheoesophageal fistulas, one had left main bronchioesophageal fistula, and one had tracheo‐mediastinal fistula.

Assessment of the modified Medical Research Council dyspnea scale (mMRC) grade, PS, symptoms before and after SEMS placement are shown in Table 2. For mMRC grade, 20 of the 24 patients (83.3%) showed improvement, there was no change in three patients, and one with exacerbation. For PS, 19 of the 24 patients (79.1%) showed improvement, there was no change in four, and one exacerbation. For symptoms, 21 of the 24 patients (87.5%) showed improvement and there were three patients with exacerbations after SEMS placement.

**Table 2:** Assessment of mMRC, PS and symptoms before and after SEMS placement

	Before SEM placement	After SEMS placement	No. of patients
mMRC	Grade 4	Grade 1	1
	Grade 4	Grade 3	2
	Grade 3	Grade 2	15
	Grade 3	Grade 3	1
	Grade 3	Grade 4	1
	Grade 2	Grade 1	2
	Grade 2	Grade 2	2
PS	PS 3	PS 1	1
	PS 3	PS 2	14
	PS 3	PS 3	4
	PS 3	PS 4	1
	PS 2	PS 1	4
Symptoms	Improvement		21
	Improvement⇒Exacerbation		2
	Exacerbation		1

mMRC, modified Medical Research Council dyspnea scale.

**Figure 1:**
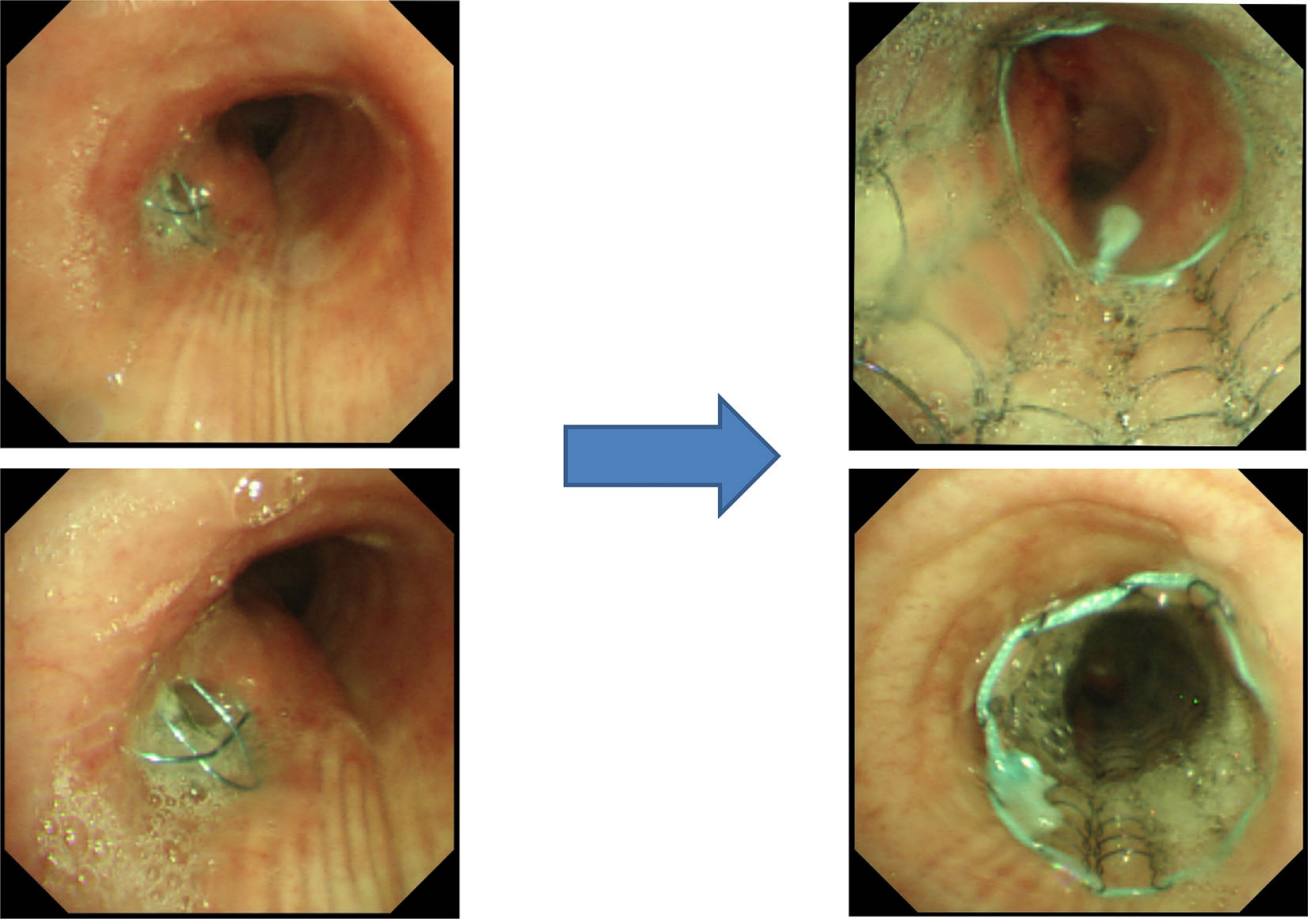
Case 1. Tracheoesophageal fistula due to esophageal cancer. Covered Ultraflex (16 × 60 mm) was inserted into the tracheoesophageal fistula. The stent had migrated into the upper portion of the trachea and was subsequently reinserted.

With regard to SEMS‐related complications, follow‐up CTs, and bronchoscopic examinations revealed that there were no patients who suffered from obstructive granulation, infection, or mucous plugs. No procedure‐related mortality was noted. However the inserted covered Ultraflex stent in three patients migrated out of place (Fig 1) and patients needed additional stent insertions; one needed additional insertion of a larger‐sized stent due to stent migration, and one needed an additional insertion of smaller‐sized stent due to stent migration after three months. The migration was associated with the small diameter of the stent, and the shape of the airway space next to the stenosis.

**Figure 2:**
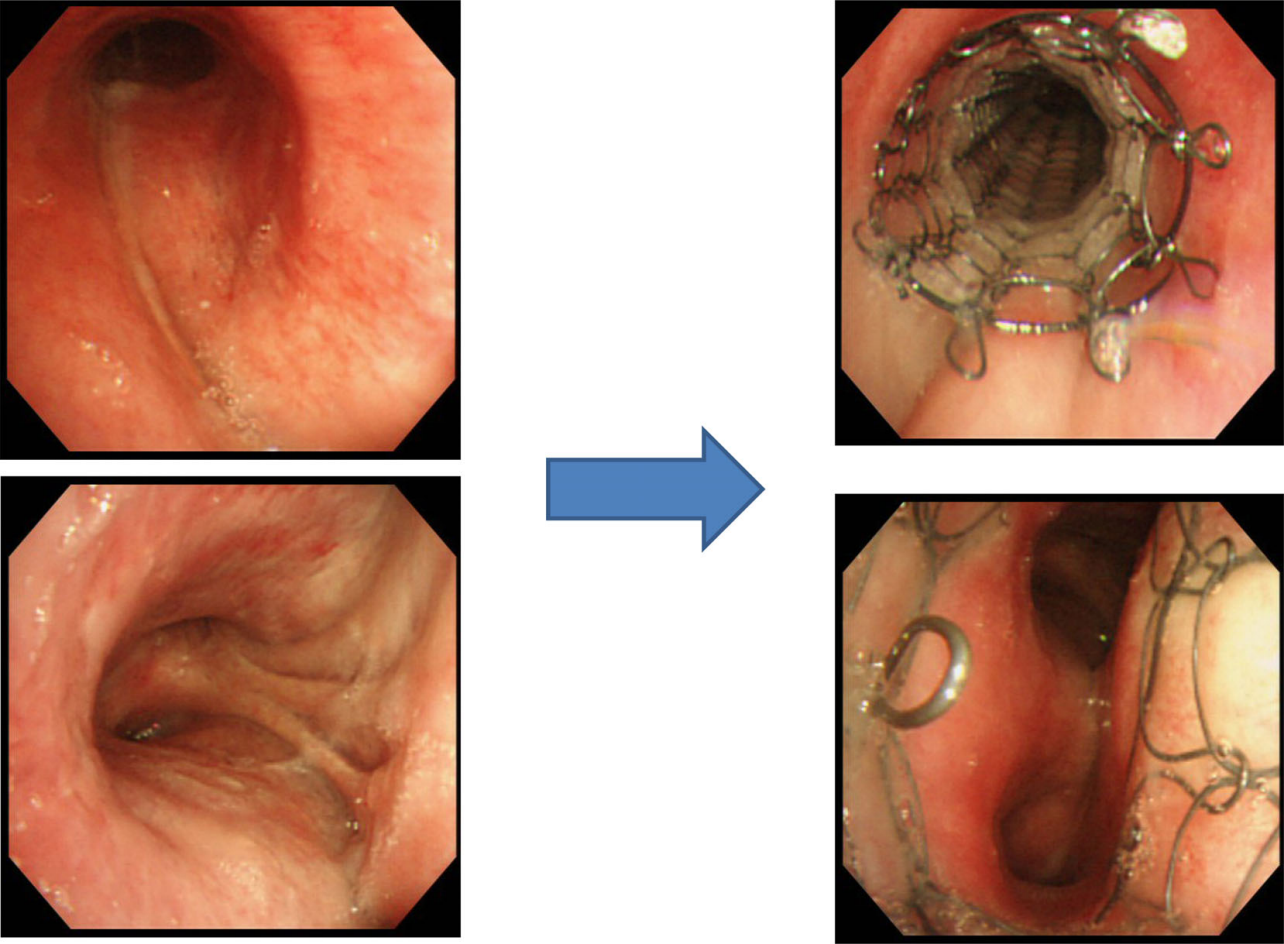
Case 2. Tracheal stenosis due to malignant lymphoma. Covered Ultraflex (10 × 60 mm) was placed into the tracheal stenosis. This patient was a longer survivor after stent placement.

**Figure 3:**
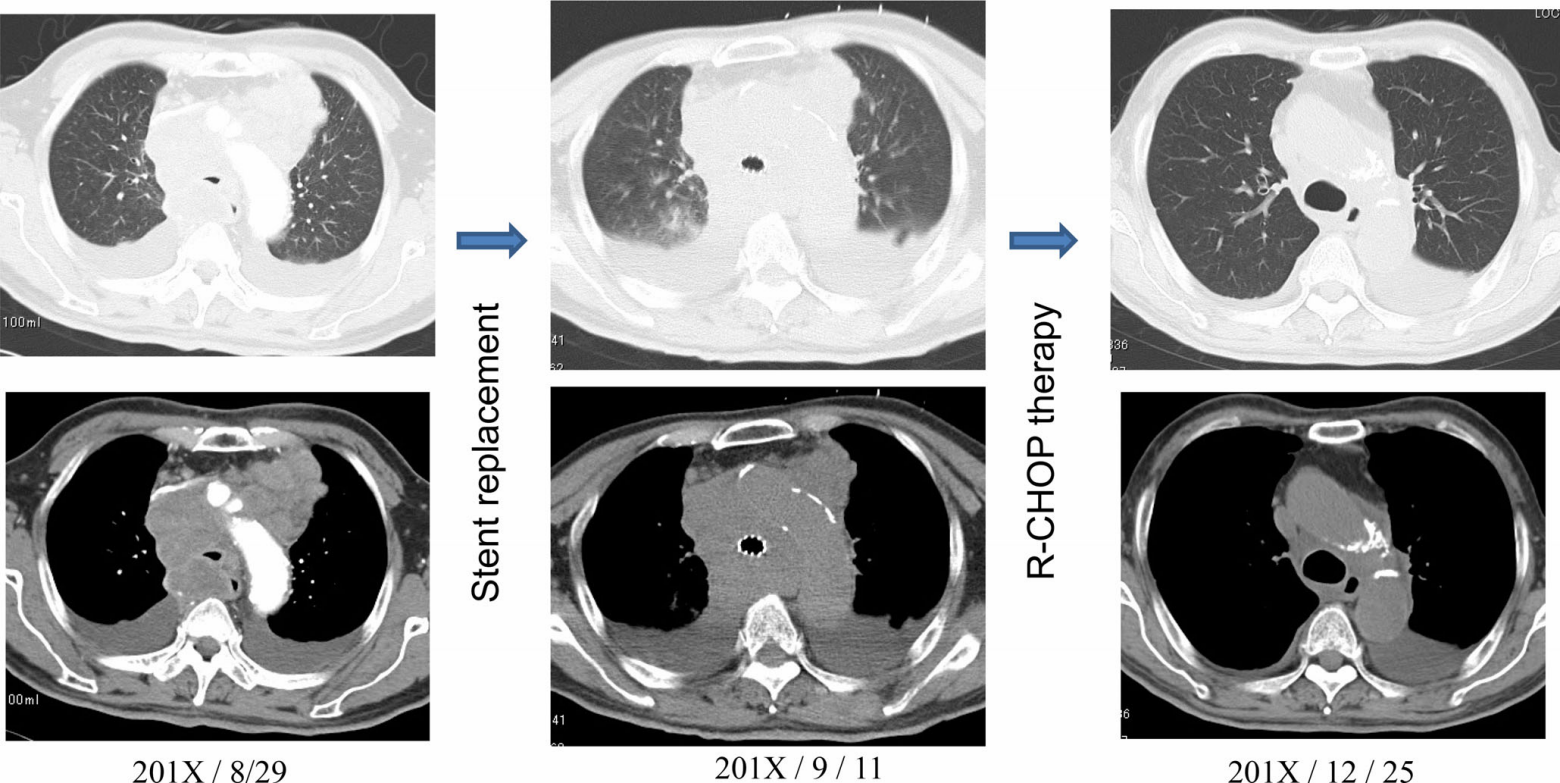
Case 2. Tracheal stenosis due to malignant lymphoma. Covered Ultraflex (10 × 60 mm) was placed into the tracheal stenosis. Pathological examination revealed that the patient had a diffuse large B cell after stent placement. R‐CHOP therapy (six times) was effective and the patient was well six years and seven months after placement.

**Figure 4:**
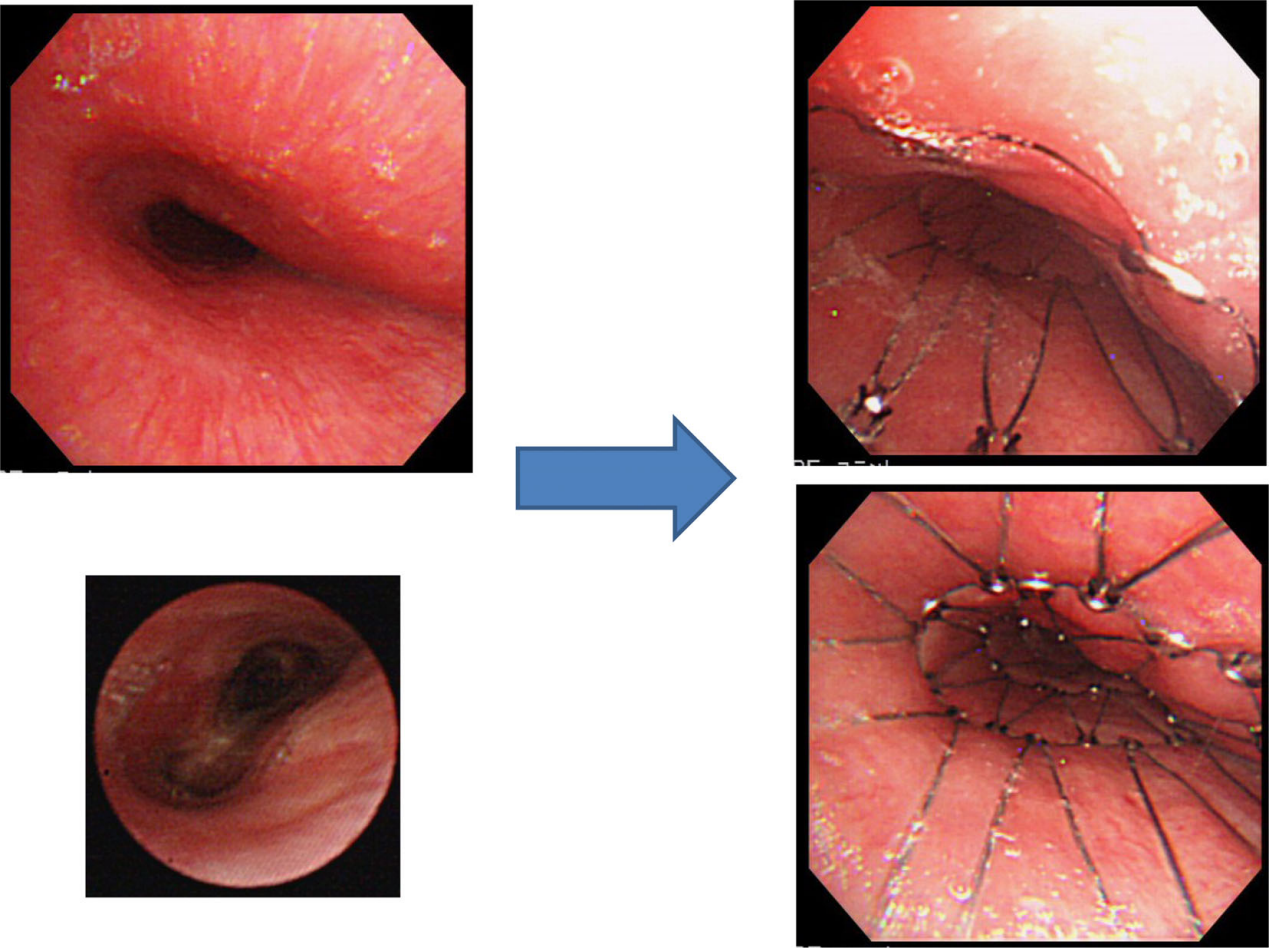
Case 3. Tracheal stenosis due to thymic cancer. A spiral Z stent (20 × 80 mm) was placed into the tracheal stenosis. This patient was a longer survivor (six years and six months) after stent placement.

**Figure 5:**
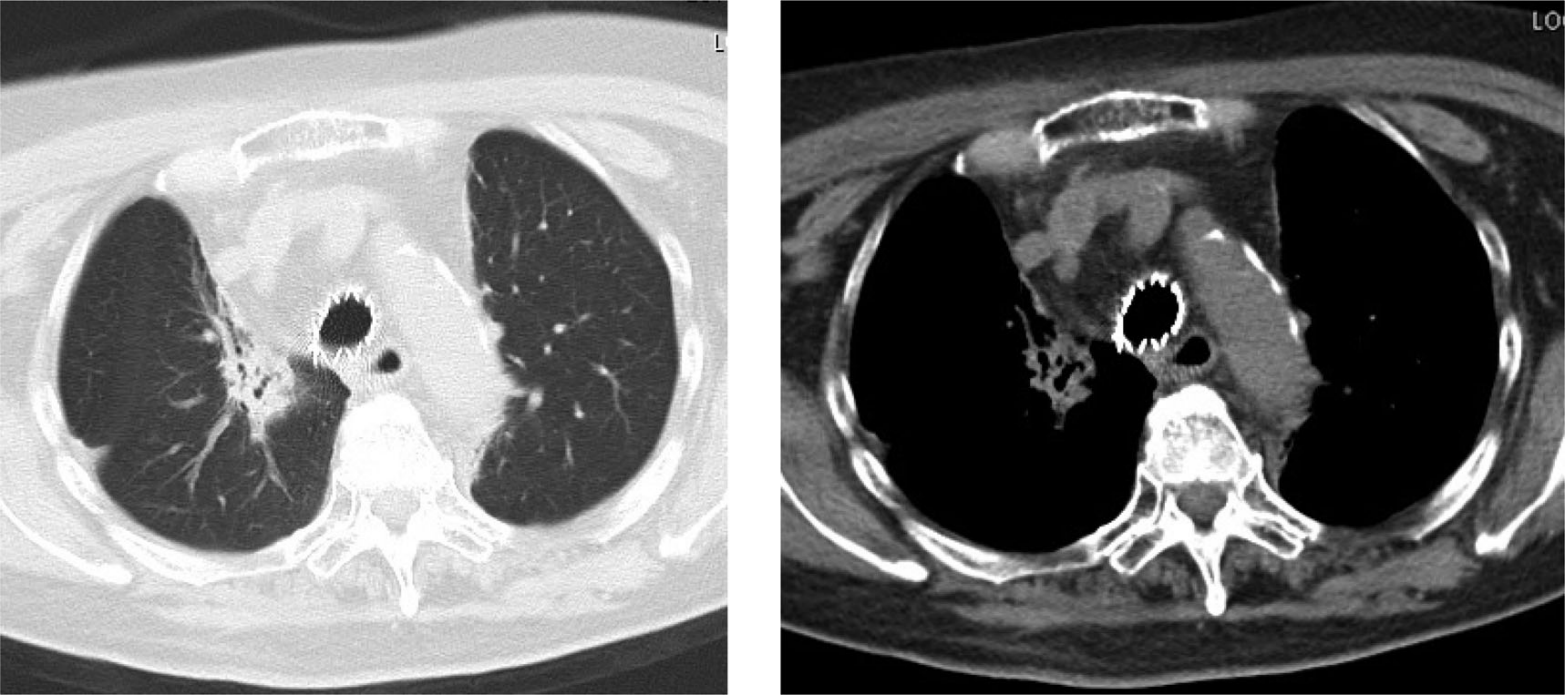
Case 3. RT 60 Gy + ADOC (CDDP+DXR + VCR + CPA) six courses were given. Chest CT five years after stent placement showed good tracheal patency.

Two patients who suffered from CAS as a result of malignant lymphoma and thymic carcinoma survived for longer periods of time after SEMS placement because the malignant diseases were also being treated with efficient therapies. The patient with malignant lymphoma lived for six years and seven months after stent placement (Figs 2, 3). The patient with thymic cancer died six years and six months after stent placement (Figs 4, 5).

**Figure 6:**
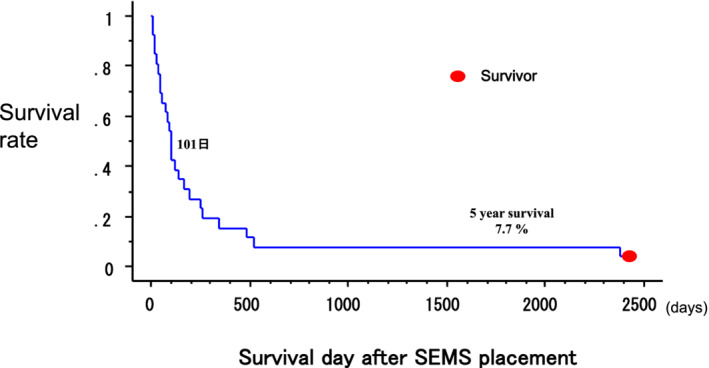
Survival rate of all the patients after SEMS placement.

Four patients with CAF died due to tumor growth within four months (45, 102, 104, and 120 days) after stent placement. Median survival after stent insertion for malignant CAS and CAF was 101 days, and mean survival was 312 ± 630 days (Table 1, Fig 6). The five‐year survival rate of all patients after SEMS placement was 7.7% (Fig 6). Median survival after stent insertion was 98 days for CAS and 103 days for CAF, and mean survival was 383 ± 707 days for CAS and 93 ± 33 days for CAF. The five‐year survival rate after stent insertion for patients with central airway stenosis was 9.1%, which was better than that of the patients with airway fistulas (0%), but there were no significant differences (*P* = 0.316). The mean survival days (250 ± 240 days) of the three patients with acute respiratory failure was not significantly shorter than that (346 ± 694 days) of the 21 patients without acute respiratory failure.

## Discussion

SEMS implantation via bronchoscopy can immediately relieve CAS and CAF, and improve quality of life (QOL) and patient survival. Improved understanding and recognition of cancer‐related symptoms can improve management strategies, patient compliance, and QOL for all patients with lung cancer.^5^ After SEMS placement, 20 patients (83.3%) showed improvement in mMRC grade, 19 (79.2%) improvement in PS, and 21 (87.5%) improvement in symptoms. A total of 21 patients (87.5%) with malignant CAS or CAF had improved mMRC grade, PS and symptoms after SEMS placement. The SEMS placement for CAS or CAF was associated with improvement in the QOL in 87.5% of the patients.

The clinical success rate of SEMSs for airway stenoses and fistulas was reported to be 93% (26/28).^6^ This treatment of patients with malignant CAS and CAF was found to be effective for QOL of patients.

The US Food and Drug Administration (FDA) advised caution in July 2005 regarding the use of metallic stents for benign airway disease.^7^, ^8^ The use of permanent SEMSs for benign tracheobronchial stenosis has also been reported to be effective and safe for the majority of patients in long‐term follow‐up.^9^ In a study by Sökücü *et al*. no significant differences in symptom palliation, insertion safety, complication rate or survival were detected between silicon and metallic stents.^10^

The common stent‐related complications of SEMSs have been reported to be secretion retention (25%, 9/36), development of granulation tissue (13.9%, 5/36), tumor in‐growth (13.9%, 5/36), and hemoptysis (8.3%, 3/36).^11^ Saad *et al*.^12^ reported that observed complications included infection (15.9%), obstructive granulomas (14.6%), and migration (4.7%): the incidence of granulomas was significantly lower in patients with lung cancer (4.0%) versus the lung transplantation and benign conditions group (17.3% and 33.3%, respectively; *P* = 0.002). In our results, stent migration (12.5%: 3/24) was more frequent than that (4.7%) reported by Saad *et al*,^12^ but infections and obstructive granulomas were not seen. The application of the “side‐by‐side” method of bilateral SEMSs to malignant carinal involvement has been reported to be less invasive and safer under topical anesthesia in all cases.^13^ Although self‐expandable bifurcated metallic stents have been reported to cause mucus plugs,^14^ we did not experience this complication. To mitigate stent migration, we chose a more suitable larger Ultraflex or added a baloon inside the stent. Metallic Y‐shaped stent placement has been shown to be technically feasible, effective and safe for a bronchopleural fistula around the upper carina.^15^ Third‐generation fully‐covered SEMSs are a safe treatment option for complex benign airway stenosis, but complications requiring stent removal have been frequently reported.^16^

It is controversial whether a patient with acute respiratory failure is a candidate or not for a stent replacement, because of a poor prognosis after stent replacement.^17^ In our research, the mean survival days (250 ± 240 days) of the three patients with acute respiratory failure was not significantly shorter than that (346 ± 694 days) of the 21 patients without acute respiratory failure. Our results showed that malignant CAS and CAF could be treated with SEMS placement, with or without acute respiratory failure.

Patients needing placement of tracheobronchial stents for neoplastic diseases are usually terminal. That being said, in our study there were two patients who were suffering from CAS as a result of malignant lymphoma and thymic carcinoma that survived longer than expected. The increase in their survival was due to the fact that the malignant diseases were treated well and controlled. Based on this retrospective study, we questioned how we could lengthen survival for these patients. Prognosis between different types of cancer is actually difficult to compare. It is expected and well known that ED SCLC has poorer prognosis in comparison with some forms of malignant lymphomas. The majority of patients with malignant CAS and CAF are reported to be in poor condition. Patients with advanced esophageal cancer and airway involvement have a poor prognosis.^17^ On the other hand, symptoms of CAO are similar no matter which type of cancer is the cause. However, we believe that it is beneficial to evaluate survival as well as QOL of the patients who have had SEMS placement.

The five‐year survival rate after stent insertion for our patients with CAS was 9.1%. The six‐month overall survival of patients with SEMS placement has been reported to be 44% (11/25).^11^ Some researchers are of the opinion that airway stenting does not improve survival but they believe it does improve QOL for patients with malignancy. We believe airway stenting improves not only QOL but also lengthens the lifespan of patients with malignancy. Our data showed that 80%–87.5% of patients treated with SEMSs had a better mMRC grade, PS, and symptoms after SEMS placement. Furthermore, in the case of the patients with malignant lymphoma and thymic carcinoma, tumor size was actually reduced after treatment and airway stenosis disappeared and both patients survived more than six years. Lung cancer patients with tracheobronchial stenosis can avoid urgent airway stenosis with SEMS placement. They will also benefit from treatment with the latest generation of drugs (EGFR‐TKI [epidermal growth factor receptor‐tyrosine kinase inhibitors]),^18^ anaplastic lymphoma kinase (ALK) inhibitors^19^ and immune checkpoint inhibitors^20^ giving them a greater chance of a longer life. Programmed death‐1 (PD‐1), as well as programmed death‐ligand 1 (PD‐L1) inhibitors for the treatment of advanced non‐small cell lung cancer (NSCLC) are currently available and have demonstrated antitumor activity.^21^ These new drugs have been reported to significantly improve the overall survival (OS), progression‐free survival (PFS), and objective response rate (ORR) in advanced NSCLC patients who are *EGFR* gene mutation positive, *ALK* gene mutation positive, or PD‐L1 expression positive. It is hoped that additional effective therapies will improve survival as well as QOL and the symptoms of the patients with CAS in the near future. Longer survival was seen in the patients who received additional therapies after airway stenting than those without further therapies (*P* < 0.01).^17^ Small cell lung cancer patients with airway stenting experienced longer survival when post‐procedural tumor‐specific therapies were performed.^22^

Malignant fistulas are associated with very short life expectancy of only a few weeks or months.^23^ In our series, the patients with tracheobronchial fistulas died within four months (45, 102, 104, and 120 days) after stent replacement, but QOL increased after stent placement: patients could eat, were able to drink and had less aspiration into their airways. Malignant tracheobronchial fistulas can also be treated by inserting a covered SEMS.^24^

Airway stenting using silicone stents has been reported to be safe and effective in palliation of airway stenosis in patients with untreated malignant lymphoma, and permits post procedural tumor‐specific therapy.^25^ No significant differences in symptom palliation, insertion safety, complication rate or survival have been detected between silicon and metallic bifurcated stents.^10^ SEMSs for CAS and CAF should therefore be considered because of less serious complications and improvement of symptoms after stent placement.

This research had some limitations. It was a single‐center study, with a small number of patients with lesions and a very long study period. Further prospective larger sample size and/or multicenter studies are needed to improve the findings of the current study.

In conclusion, 87.5% of the patients showed improvement of CAS and CAF. There were no patients who suffered from obstructive granulation, mucous plugs and no procedure‐related mortality. However the inserted covered Ultraflex stent in three patients migrated out of place and those patients needed additional stent insertions. Median survival days of patients with malignant CAS and CAF after the stent insertion was 101 days and mean survival days after the stent insertion was 352 ± 680 days. Two patients who were suffering from CAS as a result of malignant lymphoma and thymic carcinoma survived for more than six years because the malignant diseases were treated by efficient therapies. Patients with malignant CAS are usually terminal, but the possibility of increasing survival time is likely to be due to the new efficient therapies.

## Disclosure

All authors have no conflict of interest to declare.
